# Observational Methods in Studies of Infant and Young Child Feeding Practices in Low- and Middle-Income Countries: A Twenty-Year Retrospective Review

**DOI:** 10.3390/nu16020288

**Published:** 2024-01-18

**Authors:** Teresa R. Schwendler, Muzi Na, Kathleen L. Keller, Leif Jensen, Stephen R. Kodish

**Affiliations:** 1 110 Chandlee Laboratory, Department of Nutritional Sciences, The Pennsylvania State University, University Park, PA 16802, USA; 2 202 Rodney A. Erickson Food Science Building, Department of Food Science, The Pennsylvania State University, University Park, PA 16802, USA; 3 Armsby Building, Department of Agricultural Economics, Sociology, and Education, The Pennsylvania State University, University Park, PA 16802, USA; 4 219 Biobehavioral Health Building, Department of Biobehavioral Health, The Pennsylvania State University, University Park, PA 16802, USA

**Keywords:** observational methods, direct observations, indirect observations, spot checks, infant and young child feeding, low- and middle-income countries

## Abstract

This narrative review describes the observational approaches used to study infant and young child feeding (IYCF) practices in low- and middle-income countries (LMICs) published between 2001 and 2021. Articles were included in this narrative review if they were (1) original peer-reviewed articles published in English in PubMed and Web of Science; (2) published between 1 January 2001, and 31 December 2021; (3) conducted in an LMIC; and (4) employed observations and focused on IYCF practices among children aged 6–59 months. The studies (*n* = 51) revealed a wide-ranging application of direct meal and full-day observations, as well as indirect spot checks, to study IYCF. The findings revealed that meal observations were typically conducted during a midday meal using precise recording approaches such as video and aimed to understand child–caregiver interactions or specialized nutritious food (SNF) usage. Conversely, full-day observations lasted between 6 and 12 h and often used a field notes-based recording approach. Behaviors occurring outside of mealtime, such as snacking or interhousehold food sharing, were also a primary focus. Finally, spot checks were conducted to indirectly assess SNF compliance during both announced and unannounced visits. This review highlights the adaptability of observations across contexts and their versatility when used as a primary data collection tool to help monitor and evaluate nutrition programs.

## 1. Introduction

Children living in low- and middle-income countries (LMICs) disproportionally contribute to the global burden of child undernutrition and thus remain a target population for intervention [1]. The multi-level determinants of nutritional status can be conceptualized as basic, underlying, and immediate, depending on their proximity to nutrition outcomes [2]. One immediate cause of undernutrition is a suboptimal diet [2]. The dietary intake of children is often reliant on the feeding behaviors of their caregivers. Thus, understanding those behaviors, and the factors that influence them in any given setting, is important for researchers and practitioners aiming to develop or evaluate nutrition programming. 

To do so, researchers have historically utilized observational methods drawn from the field of anthropology to examine and understand feeding and dietary behaviors [3]. There are four primary reasons for using observations to study young child feeding. First, observations allow researchers to understand the order in which behaviors occur. There is a complex series of behaviors involved in infant and young child feeding (IYCF) practices (i.e., food preparation, intrahousehold food allocation, young child–caregiver interaction, food storage and re-consumption, etc.), which requires an in-depth understanding of the unique behavioral chain of feeding behaviors to a given context [4,5,6]. Second, other assessment methods that are reliant on recall, such as surveys, have limited usefulness if the researcher does not first have a formative understanding of the current behaviors in question. Third, observations allow for understanding IYC responses to caregiver actions given that other methods that involve self-report are not a viable option for IYC. Fourth, in behavioral research, what people say and what they do may be at odds, especially in contexts where social desirability bias may be a risk to the validity of findings [7]. This can occur when the norm is to provide socially desirable responses for fear of losing social protection services or aid support [7]. As Bernard [3] states: “When you want to know what people actually do there is no substitute for watching them or studying the physical traces their behavior leaves behind”. Observations have thus been an important method used to study IYCF behaviors across settings. 

Bernard describes two fundamental types of observational methods that explore human behavior: direct and indirect observations [3]. The study of IYCF behaviors lends itself to both. Direct observations allow for documentation of behaviors in real time, for example, when an observer will record behaviors of interest while watching a household during a mealtime [3]. During direct observations, an event (i.e., behavioral codes are assigned to events), an interval (i.e., behavioral codes are assigned to a time interval), or a combination of these approaches is adopted for recording behaviors [8]. By contrast, indirect observations evaluate human behaviors through shadowed data, which offer indirect evidence of behavior [3]. A common indirect approach is a spot check, which includes an unannounced visit from an observer to a study participant. Using spot checks to assess daily supplement compliance by comparing the number of supplements that a participant has available based on the number that should be available with perfect compliance is one application of this approach [9]. Direct and indirect observations can take other forms as well. 

To our knowledge, no review has critically examined observational methods used to study IYCF practices across LMICs. One review, conducted in 2011, focused on summarizing responsive feeding measures, both observational and self-reported, but the results were focused on the relationship between responsive feeding measures and child outcomes instead of the observational approaches employed [10]. Given the wide range of IYCF behaviors that can be explored using observations, our review seeks to broadly build on this previous work. We conducted this review (1) to explore what observational approaches have been used to study IYCF practices in LMICs between 2001 and 2021 and (2) to describe specific methods, recording approaches, and analytic procedures used within those approaches.

## 2. Materials and Methods

This narrative review sought to synthesize the published peer-reviewed literature that used observations to understand IYCF practices across LMICs between 2001 and 2021. This review followed the narrative review standards set by Torraco, including adhering to a methodological explanatory structure that highlights review findings by method [11]. This approach is typically used when multiple methodologies (i.e., different types of behaviors, observational methods, recording approaches, and analytic procedures) are simultaneously being used to study the same well-established topic, for instance, IYCF behaviors. 

### 2.1. Search Strategy

The primary author, TRS, met with a health sciences librarian to establish and refine key search terms. Keyword searches were conducted by TRS in PubMed and Web of Science using a combination of the following terms: observation, direct observation, household observations, spot check, unstructured, structured, semi-structured, infant, and young child diet, child caregiver interaction, child, diet, feeding, region, and low/middle income. Throughout the process, TRS used Excel to track the articles retrieved from each keyword search and prevent redundancies. 

### 2.2. Inclusion and Exclusion

Studies were limited to original peer-reviewed journal articles that were published in English from 2001 to 2021. Articles were first screened by title and excluded if they were unrelated to the study topic; all other articles were screened at the level of abstract by TRS. Articles were retained if they were conducted in an LMIC as defined by the World Bank in 2021, employed direct or indirect observations, provided a description of the observational methods, and focused on IYCF practices among children aged 6–59 months [12]. Observations could focus on the child or the household at large but had to include target behaviors related to IYCF. Observations that only focused on related behavioral domains that are often included in nutrition programming, such as water, sanitation, and hygiene (WASH), care, child development, nutrition education, breastfeeding, health counseling, or the household environment, but did not include an explicit focus on IYCF, were excluded. Articles were also excluded if the description of the observational approach was insufficient to determine the type or employed analysis approach. 

### 2.3. Data Extraction and Synthesis Technique 

The final articles were read in their entirety by the primary author, TRS, and information on each study objective, research design, age of observed infant or child, type of observational method(s), target behaviors, behavioral coding method, and analytic approach were recorded. Findings were then summarized in tabular and narrative forms by TRS and SRK. The solicitation of articles, data extraction, and summarizing of findings occurred between 2021 and 2022. 

## 3. Results

**Figure 1:**
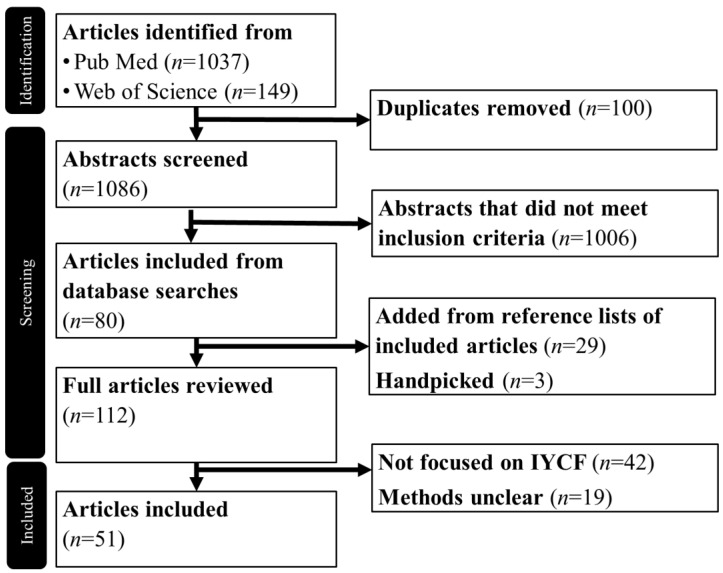
The process used for the identification of articles.

In total, 1086 titles were screened for inclusion, 112 articles were reviewed in full, and 51 articles were included in this review (Figure 1).

The studies included in this review report on observational findings across multiple regions, including Sub-Saharan Africa (28), Latin America and the Caribbean (4), South Asia (14), Southeast Asia (4), and one multi-site study. The studies employed both direct and indirect observations to study IYCF.

### 3.1. Direct Observations

Direct observations were the most frequently reported approach to study IYCF practices from 2001 to 2021, including both meal and full-day observations. 

#### 3.1.1. Meal Observations

##### Overview

Direct meal observations explicitly focused on one meal, consisting of one or more feeding/eating episodes. Most studies did not define a “meal”, but we considered the consumption of food items or the consumption of specialized nutritious food (SNF) as a “meal” in this analysis [13,14,15,16,17]. Observations that did not specify a meal but were <5 h were also considered meal observation in this review [18,19,20,21]. In addition to behaviors during mealtime, those occurring both before (e.g., washing utensils during meal preparation) and after (e.g., storage of leftovers) meals were also reported in studies to answer broader questions related to feeding context [18,21,22,23,24,25,26,27,28,29]. Meal observations were sometimes conducted during the first meal of the day [30,31] but more frequently during a midday meal [13,16,22,26,32,33,34,35,36,37]. Some researchers observed not just one meal/day but all meals throughout the day (i.e., morning, midday, and evening meals) while sampling from different households or by arriving at the same household on different days [18,19,23,24,38,39]. Others observed timed eating episodes [14,15,40,41,42] or a meal during an unspecified time of day [17,20,21,25,27,28,29] (Table 1). 

##### Objectives of Meal Observations

Meal observations primarily explored caregiver interactions with children during meals outside the scope of interventions [13,16,18,19,20,22,25,26,30,31,34,35,37,39]. Three studies assessed changes in mother–child dyad behaviors between exposure and control participants following an intervention [32,33,36]. Direct observations were also used to assess SNF compliance and consumption patterns during mealtimes, including those focused on lipid-based nutrient supplements (LNSs), micronutrient powders (MNPs), and locally prepared supplemental foods [14,16,22,23,24,28,29,31,38]. By contrast, controlled meal tests were sometimes designed to assess SNF acceptability outside of usual mealtimes [14,15,38,40,41,42]. 

Meal observations have also been used in formative studies to more broadly understand IYCF practices for informing intervention design and implementation [17,21,22,23,24,28,29,31]. An ethnographic study in Kiribati, for example, used them in combination with other methods to describe the ecological factors influencing IYC diets [27]. In one instance from Nepal, visual food intake estimates made during meals were used to validate actual intake based on food weights [17]. 

##### Behavioral Recording Approaches of Meal Observations

*Event and interval based.* Several studies used a combination of event- and interval-based approaches for recording target behaviors as they occurred, for example, every 5–10 min during a meal [17,23,24,27,43]. The combined approach involved recording each instance of a target behavior as it occurred [23,24,27,43], and in cases of continuous behaviors (e.g., the caregiver was cleaning pots), actions were recorded only every so many seconds or minutes [23,24,27,43]. Structured behavioral checklists were an alternative tool used to record target behaviors observed during 30-s intervals in two studies [18,19].

*Event based only.* Caregiver–child interactions were often recoded using an event based approach [13,21,22,25,28,31,32,33,34,35,36,37,39]. For instance, a study in India recorded target IYC interest in food, social interactions during the meal, child self-feeding, and caregiver encouragement, among others, when they occurred ≥3 times during a mealtime [25]. A study in Cambodia took a similar recording approach to mealtime behaviors but only if they occurred ≥1 time [13]. Feeding-related behaviors observed at each intended bite (i.e., each time a spoonful of food was offered to the child) were recorded in six studies conducted across Ethiopia, Bangladesh, Malawi, and Vietnam [30,31,32,33,34,35,36,37,39]. 

*Interval based only.* Eight studies recorded mealtime behaviors (e.g., caregiver–child interactions) within a set time interval, for example, all of those occurring during a 5 min time window [14,15,16,26,38,40,41,42]. For example, in India, Malawi, Ghana, and Burkina Faso, researchers used timed meals to assess SNF acceptability by reporting the amount consumed during a specified time interval [14,38,40,41,42]. Observations conducted in Peru and India also assessed child liking of novel nutritional supplements using a five-point hedonic scale during a timed SNF meal [14,15]. 

Actual recording approaches varied considerably: ten studies used a structured form or checklist [14,17,18,19,23,24,25,27,32,33,36], six used videotaping [15,30,34,35,37], and one required detailed field notes [21]. Several studies did not report recording approaches [16,17,20,22,26,28,29,38,39,40,41,42]. 

##### Analytic Approaches to Meal Observations

*Statistical analysis.* Most meal observations found in this review were analyzed statistically by comparing the consumption of different test meals in grams or the proportion of the total consumed supplement [14,15,38,40,41,42], assessing differences in SNF acceptability using a hedonic scale [14,15], or calculating the proportion of target behaviors observed every 5 min [16,26]. In addition, associations between several studies assessed relationships between caregiver care practices or feeding styles and child eating behaviors [30,31,34,35,37,39], including studies in Malawi and Vietnam that reported the odds of child bite acceptance under different feeding styles and meal conditions [31,34,35]. A responsive feeding intervention in Bangladesh used pre- and post-measures to determine its impact on caregiver feeding style [32,33,36]. Responsive feeding scales were validated in observational studies conducted in Cambodia and India as well [13,25].

*Textual analysis.* In several studies, textual data from meal observations were analyzed thematically to understand behavioral patterns reflective of SNF utilization [22,23,24,28,29,43]. Only one study explicitly described using a theory-based approach to guide textual analysis, for instance, by using existing constructs from established behavioral models in deductive coding [27]. 

#### 3.1.2. Full-Day Observations

##### Overview

Direct observations conducted across multiple meals during a single day were considered “full-day” observations for purposes of this review. Studies using full-day observations ranged between 5 and 9 h [44,45,46,47], 10 h [48,49], 11 h [50], and ≥12 h long [51,52,53,54,55,56] or between sunrise and sunset [23,24]. In four studies, the duration was unreported [57,58,59,60,61] (Table 2).

##### Objectives of Full-Day Observations 

Approximately half of the studies in this review utilized full-day observations to specifically examine one or more aspects of SNF compliance beyond a single mealtime, often while also studying other IYCF behaviors [23,24,47,49,50,52,53,55,57,61]. The other half used full-day observations focused not on supplements but on a wider range of IYCF practices, including breastmilk consumption, complementary food intake, care practices, and food preparation patterns, as well as underlying contextual factors (e.g., intrahousehold food sharing, gender dynamics) [27,44,45,46,48,51,54,56,58,59,60].

##### Behavioral Recording Approaches of Full-Day Observations

*Event and interval based.* In four studies, researchers observed behaviors every 5–10 min at minimum, with ad hoc recording when those specific to the study aims occurred [23,24,27,53]. In Burkina Faso, researchers recorded target behaviors guided by a 30-min interval grid [52]. 

*Event based only.* Four studies continuously recorded all IYCF-related behaviors during the reviewed full-day observations [44,45,58,61], while another five studies specifically focused on SNF consumption patterns [47,49,50,55,57]. Care during mealtime, rather than dietary practices, was the specific focus of observations conducted in Kenya and Gabon [48,60]. 

*Interval based only.* Just four studies used an interval-based approach during full-day observations, including measurement of food weights just before and immediately following mealtimes throughout the day [51,54,56,59].

Among all full-day observations reviewed, thirteen studies recorded behaviors using a semi-structured form or checklist [23,24,27,46,47,48,49,50,52,55,57,60], four used detailed field notes [44,45,58,61], one used videotaping [45], and one used a personal digital assistant [53]. Four studies did not report an approach [51,54,56,59]. 

##### Analytic Approaches of Full-Day Observations 

*Statistical analysis.* Data from full-day observations were primarily analyzed statistically. In studies where SNFs were evaluated, researchers made comparisons between trial arms. Studies conducted in Bangladesh and Malawi assessed the difference in intake of supplements by the intended beneficiary, while studies in Burkina Faso and Malawi compared consumption between intended beneficiaries and other household members [50,52,53,55,56]. A study conducted in Mexico assessed the proportion of households carrying out behaviors related to key messages promoting supplement compliance [47]. A wide range of behavioral variables were compared across supplement acceptability and compliance studies. 

In studies where IYC diets were assessed, without SNFs, the analysis focused on estimating the actual consumption of breastmilk or complementary foods by reporting food weights to estimate the proportions of foods consumed [46,51,54,59]. Studies conducted in India and Malawi validated intake estimation techniques comparing observational findings with weighted food records [46,54]. A study in Bangladesh assessed the adequacy of infant nutrient and energy intakes over a 12 h period [51]. 

Three studies analyzed childcare and feeding indicators. A study in Kenya assessed the differences in sociodemographic variables and participant time spent carrying out childcare behaviors [48]. A study conducted in Gabon used observation and survey data to create an index of access to care, food, health, and natural resources, which was then assessed against child anthropometry [48,60]. Several full-day studies assessed associations between observed behaviors and anthropometric or biomarker indicators of IYCs [48,50,53,56,60]. 

*Textual analysis.* In Malawi and Mozambique, researchers thematically analyzed event- and interval-based textual data to understand SQ-LNS utilization at household and community levels [23,24,43]. Similar analytic approaches were used to assess MNP compliance in Bangladesh, specifically [57]. A wide range of nutrition-related practices around mealtimes were assessed using inductive analyses of textual data from behavioral codes in Indonesia and Bangladesh [45,58]. In Mexico and Kiribati, researchers took a deductive approach to guide content analysis [27,44]. Finally, a formative study in Zanzibar explored maternal perceptions toward an instant soy–rice supplement with and without milk powder, as well as the proportion of caregivers preparing the supplement correctly and differences in child consumption patterns [61]. 

### 3.2. Indirect Observations

#### 3.2.1. Spot Checks

##### Overview

The only type of indirect observation found in this review was spot checking. Spot checks, in these studies, involved an enumerator visiting a participant’s home (announced or unannounced) and checking for shadowed data reflective of the behavior of interest (Table 3).

##### Objectives of Spot Checks

We reviewed six studies that used spot checks to gauge SNF compliance [9,28,42,43,53,62]. In Burkina Faso, spot checks during home visits assessed IYC (9–18 mo.) adherence to SQ-LNS and dispersible zinc tablets [53]. Unannounced spot checks were conducted in Niger over four weeks to assess RUTF utilization among households with children with severe acute malnutrition [9]. Compliance with a novel SQ-LNS supplement was assessed similarly but over 8 weeks in Mozambique [43]. Finally, spot checks were used in Bangladesh to understand how different distribution modalities impacted MNP compliance [62]. 

##### Behavioral Recording Approaches of Spot Checks

Six unannounced spot checks in all contexts were conducted by counting the number of unused versus used supplements [9,28,42,43,53,62]. 

##### Analytic Approaches to Spot Checks

*Statistical analysis.* The data from the spot checks were analyzed using simple descriptive statistics focusing on understanding supplement compliance. For example, weekly deviance from an LNS distribution regime was calculated by comparing the observed sachets available during spot checks with those that should have been available given expected consumption among IYC in rural Niger [9]. Studies in Burkina Faso, Ghana, Mozambique, Bangladesh, and Kenya took similar analytic approaches [28,42,43,53,62]. The findings from Burkina Faso were reported as a daily disappearance rate by dividing by the number of observation days [53]. 

## 4. Discussion

This review of studies employing observational methods to study IYCF practices between 2001 and 2021 built on the work of Bentley and colleagues, who conducted a similar review in 2011 [10]. We found 51 relevant nutrition studies published in LMICs during this time, including 28 from Sub-Saharan Africa, 4 from Latin America and the Caribbean, 14 from South Asia, 4 from Southeast Asia, and 1 multi-site study. Most frequently, direct observations were utilized to understand IYCF practices during mealtimes, including SNF utilization. Overall, studies using full-day observations shared similar objectives to those of meal observations but had the benefit of capturing between-meal behaviors such as snacking, in addition to contextual factors influencing IYCF practices. Full-day observations were often the method of choice when the study sought to understand IYC intake both during and between meals. Those studies that used indirect observations, namely, spot checks, did so to gauge SNF compliance. Specific considerations for using observations to study IYCF in LMICs are described below. 

Determining the specific type of observation to use during IYCF research or programs should first be determined by the type of data needed to answer the guiding research questions. Our review findings suggest that direct observations are versatile and thus can be used for exploring or understanding a wide range of IYCF practices, including supplement utilization, across the full program cycle, including before (formative [22,23,24,43]), during (process [29,53,57]), and after (summative [50,53]) interventions. By contrast, indirect observations have a narrower utility in nutrition programs as this review found them primarily used when gauging user compliance with nutrition supplements [9,28,42,43,53,62]. In both cases, textual and numerical data may be generated to explain what feeding-related behaviors are occurring, as well as why and how they are carried out in a particular setting. Observations, therefore, offer a useful mixed-methods option for those wishing to study, monitor, or evaluate feeding practices across contexts. 

Second, decisions regarding the type of observation to use in a study must be made in light of feasibility-related constraints, for example, budget, timeline, and personnel, just to name a few. Conducting full-day observations from sunrise to sunset may be optimal in many cases but unfeasible especially in the context of humanitarian and development settings where travel restrictions, coupled with far travel distances, are commonplace. Meal observations conducted in Kenya, which focused on just a 10-min meal episode, took an average of 2–3 h for preparatory work, inclusive of recruitment, completion, and ancillary measurements such as anthropometry [16]. If full-day observations are not feasible given logistical constraints, assessing behaviors that typically occur outside of usual mealtimes, such as snacking, may only be feasible using adapted approaches. For instance, conducting a series of shorter-length observations that span multiple meals across several days or weeks, but not a full day, may be a practical research compromise. Spot checks are another alternative to full-day observations when studying supplement compliance, a practice that does not always occur during an established mealtime. However, spot checks rely on shadowed data to understand behaviors and thus leave more room for measurement errors [53]. Choosing one observational method over another requires consideration of unique pros and cons that should be weighed in light of the guiding study objectives. 

Choosing the type of observational recording approach should also be primarily determined by the type of data needed to answer research questions. Recording approaches during observations in this review were typically either interval based or event based. Those that used interval-based approaches focused on specific variables usually reflective of continuous rather than one-time behaviors [63]. In the reviewed studies, interval based recording approaches were used to assess food intake throughout a meal, differences in intake between two supplements, or to validate a 24-h dietary recall [14,41,42,51,54,59]. By contrast, event based recording schemes, which may be more labor intensive when a wide range of behaviors are studied, were used to capture the behavioral frequency or sequence [63]. In cases when target behaviors are frequent and occur close together (e.g., studies of feeding styles), both interval- and event based approaches may be aided by using video to capture details more difficult to record in real-time [15,31,37]. In some cultural contexts, using video within a household may not be appropriate, however. Regardless of the chosen recording approach, building in ample planning time to refine operational variables (e.g., recording at the level of bite, snack, or meal), train observers, pilot test approaches, and ensure inter-coder reliability will help ensure data quality [63,64]. 

Various options exist for analyzing observational data, depending on the research questions, type of observation, and nature of the recorded data. Statistical approaches were used in studies where the frequencies of behaviors were of interest, such as the number of times caregivers verbally encouraged their child to eat [13,25,30,32,33,34,35,36,37,39]. For more complicated complex designs, such as those with repeated measures or those involving a large number of target behaviors, behavioral frequencies can be averaged [37], integrated into a scale or index [13,25,60], or analyzed using multi-variate models [53]. Observational findings can also be analyzed qualitatively. The textual analysis of observational field notes allows for understanding what IYCF practices occur but also why and how they occur. Theoretical frameworks, such as the socio-ecological model or biocultural framework, may be used for more deductive analysis procedures, such as content analysis [65,66]. Inductive approaches drawing from principles of grounded theory may also be applied to textual datasets when understanding new behavioral processes or building theory without a priori hypotheses are objectives [43,67].

This review was not conducted without limitations. First, only the PubMed and Web of Science databases were used to search for relevant literature, and as a result, articles not accessible through these databases may have been missed. However, we followed established search procedures used by other researchers in the field and are confident in the final set of articles included in this review [10]. Second, only the primary author was responsible for conducting the article screening process, which may have resulted in inadvertent exclusion or bias during the article selection process [68]. However, an extensive set of inclusion and exclusion criteria were established prior to the start of the review, and the selection process was well documented to decrease errors during screening [11].

Despite these limitations, in contrast to a prior review that concentrated on responsive feeding behaviors exclusively, this review explores the application of observations for various IYCF behaviors [10]. Another strength of this review was the study team who has decades of combined experience using diverse observational methods to study child eating behaviors in both laboratory and field settings [23,69,70,71,72]. We believe that this level of expertise aided in the synthesis and interpretation of findings.

## 5. Conclusions

Between 2001 and 2021, 51 published studies investigating IYCF practices in LMIC contexts revealed a wide range of applications and just as many considerations for using observational methods. Direct observations, including meal and full-day observations, have greater utility for studying a range of IYCF behaviors (e.g., dietary intake, contextual influencers, IYC–caregiver interaction, and SNF intake), while indirect observations, such as spot checks, are primarily limited to understanding SNF utilization. However, both direct and indirect observations have diverse utility while conducting formative, process, and summative evaluations of IYCF-related interventions, as well as during nutrition program monitoring. Observational methods are a useful tool with diverse applications that nutrition researchers and practitioners may use to aid in their comprehensive understanding of IYCF-related practices, which are challenging to study through self-report alone. 

## Figures and Tables

**Table 1:** Studies employing direct meal observations to study infant and young child feeding behaviors in LMICs.

Primary Author	Country	Child Age	Target Behaviors ^1^	Duration	Behavioral Recording Approaches	Analytic Approaches
Robert [22]	Tanzania, Kenya, Bangladesh	6–23	Meal: Food preparation Child and caregiver: Caregiver–child interaction, food consumption, breastfeeding, feeding behaviors, animal source food, and MNP ^2^ intake	Meal	Event	Qualitative
Kodish [24]	Mozambique	6–23	Meal: Food preparation and household food allocation Child and caregiver: Feeding behavior and intake of SQ-LNS ^3^	Meal	Event– interval based	Qualitative
Kodish [23]	Malawi	6–23	Meal: Food preparation, favoritism, child feeding, SQ-LNS ^3^ use	Meal	Event– interval based	Qualitative
Kodish [43]	Malawi and Mozambique	6–23	Child: Feeding behaviors including SQ-LNS ^3^ Caregiver: SQ-LNS ^3^ use and interhousehold and intrahousehold allocation	Meal	Event– interval based	Qualitative
Hess [40]	Burkina Faso	9–15	Child: Supplement (20 g LNS ^4^ with 0 or 10 mg of zinc) consumption during 3 days of test meals	Meal	Interval based	Quantitative
Boucheron [25]	India	12	Child: Interest in food, social interaction, volume of food consumed (using a standard stainless-steel 160 mL bowl as a reference), and self-feeding Caregiver: Verbal and behavioral encouragement, reacting to the child, harshness, social interaction, distraction, attention, who ends the meal, and hygiene practices	Meal	Event based	Quantitative
Sall [13]	Cambodia	6–23	Caregiver and child: Responsive feeding, active feeding, self-feeding, distracting feeding situation, each coded as positive (promoted food intake), neutral (no impact on intake), or negative (hindered food intake), and responses to child refusals	Meal	Event based	Quantitative
Aboud [33]	Bangladesh	12–24	Child: Bites accepted, percent of self-fed mouthfuls, and bite refusals Caregiver: Responsive acts, non-responsive encouragement acts, forceful or threatening acts, and types of foods fed to the child	Meal	Event based	Quantitative
Aboud [32]	Bangladesh	8–20	Child: Self-fed mouthfuls, bite refusals, bites accepted, handwashing, and types of foods consumed Caregiver: Responsive verbalizations, nonresponsive encouragement, nonresponsive verbalization, responsive feeding position, and type of food offered	Meal	Event based	Quantitative
Aboud [36]	Bangladesh	8–20	Child: Self-fed mouthfuls, accepted bites, refused bites, and handwashing Caregiver: Bites offered, responsive talk, directive talk, and verbal responsiveness	Meal	Event based	Quantitative
Dube [14]	India	6–36	Child: Khichri ^5^ and RUTF ^6^ consumption (grams) and child’s response to the supplement: (1) accepted eagerly, (2) accepted but not eagerly, or (3) not consumed	Meal	Interval based	Quantitative
Pachón [15]	Peru	6–11	Child: Coded child acceptance of different porridges (lyophilized meat powder and iron-fortified wheat flour) on a scale of 1 (infant consistently rejects) to 5 (infant always accepts)	Meal	Interval based	Quantitative
Abebe [37]	Ethiopia	12–23	Caregiver and child: Self-feeding, responsiveness, active feeding, social behavior, and distraction, each coded as positive (promoted feeding) or negative (interrupted feeding), and who ended the feeding episode	Meal	Event based	Quantitative
Mwase [26]	Kenya	6–24	Meal: Type of food served, plate sharing, served additional servings, and who ended the feeding episode Child: Interest in food, mood, distraction level during meal, self-feeding, and amount of food consumed Caregiver: Encouragement to eat and level of distraction	Meal	Interval based	Quantitative
Mutoro [16]	Kenya	6–24	Child: Level of interest in food, distraction, mood, and food consumed Caregiver: Encouragement during meals and distraction	Meal	Interval based	Quantitative
Baye [30]	Ethiopia	9–11	Child: Intake of standard food (Cerifam ^7^) Caregiver and child: Self-feeding, responsive, active, social, distraction, all coded as positive or negative behaviors	Meal	Event– interval based	Quantitative
Ha [34]	Vietnam	12–18	Meal: The duration of the feeding episode, foods served to the child, food consistency, utensils used to feed the child, distance between child and caregiver, who fed the child Caregiver: Physical help, verbalization, number of bites offered Child: Interest, acceptance, and rejection of bites	Meal	Event based	Quantitative
Dearden [35]	Vietnam	12–17	Meal: Consistency of food, utensils used to feed, and the person responsible for feeding Child: Bites accepted and rejected, physical actions, verbalizations, position during feeding, and self-feeding Caregiver: Verbalization and physical actions	Meal	Event based	Quantitative
Moore [39]	Bangladesh	8–24	Child: Accepted and refused bites Caregiver: Strategies to overcome poor appetite and foods offered Caregiver and child: Self-feeding, responsiveness, active feeding, social behavior, and distraction, classified as positive (promoted consumption) or negative (interrupted consumption)	Meal	Event based	Quantitative
Shankar [17]	Nepal	12–120	Child: Estimation of food portions consumed during shared plate and individual plate eating episode	Meal	Event– interval based	Quantitative
Kodish [27]	Kiribati	6–23	Meal: Food preparation, food use, and household food allocation Caregiver and child: Child feeding	Meal	Event– interval based	Qualitative
Jefferds [28]	Kenya	6–59	Meal: Food preparation with sprinkles and the number of sprinkle packets used	Meal	Event based	Qualitative
Kodish [29]	Kenya	>6	Meal: Food preparation Child and caregiver: Feeding behaviors and MNP ^2^ use	Meal	NR ^8^	Qualitative
Fouts [18]	Central African Republic	18–59	Child: Visual orientation, child state, child attachment behaviors, social behaviors, nursing and feeding behaviors, and physical location Caregiver: Response to child fussiness or crying and physical location	Meal	Event– interval based	Quantitative
Fouts [19]	Central African Republic	24–48	Meal: Who was responsible for feeding, kin relationship between feeder and child Child: Food consumed and self-feeding	Meal	Event– interval based	Quantitative
Saldan [20]	Brazil	<24	Child and caregiver: Caregiver–child interactions during feeding	Meal	NR ^8^	Qualitative
Williams [21]	Rwanda	6–59	Child and caregiver: The interactions between the child and caregiver including distractions, types, and amount of food given and hygiene-related behaviors	Meal	Event based	Qualitative
Mouquet-Rivier [38]	Burkina Faso	6–20	Child: Intake of a traditional fermented millet gruel (grams) and the improved fermented millet gruel, both standardized foods provided to study participants	Meal	Interval based	Quantitative
Phuka [41]	Malawi	8–12	Child: Intake of new lipid-based supplements vs. Nutributter^® 9^	Meal	Interval based	Quantitative
Flax [31]	Malawi	6–17	Meal: Who fed the child, type, and consistency of food Child: Interest in food, position, physical actions, verbal actions, number of bites of LNS vs. local complementary food Caregiver: Physical and verbal actions	Meal	Event based	Quantitative
Adu-Afarwuah [42]	Ghana	6–12	Child: Amount of the test meal consumed: LNS ^4^-20M vs. Nutributter^® 9^	Meal	Interval based	Quantitative

^1^ Target behaviors: The list of key target behaviors provided in this table is not exhaustive but contains key highlighted behaviors from the manuscript. ^2^ MNP: multiple micronutrient powder. ^3^ SQ-LNS: small quantity lipid nutrient supplements. ^4^ LNS: lipid nutrient supplements. ^5^ Khichri: local treatment for mild-acute malnutrition. ^6^ RUTF: ready to use therapeutic foods. ^7^ Cerifam: infant porridge provided as standardized food. ^8^ NR: not reported within the article. ^9^ Nutributter^®^: ready-to-use nutritional supplement.

**Table 2:** Studies employing direct full-day observations to study IYCF practices in LMICs.

Primary Author	Country	Child Age	Key Target Behaviors ^1^	Duration	Behavioral Recording Approaches	Analytic Approaches
Abbeddou [53]	Burkina Faso	11–16	Child: SQ-LNS ^2^ (with Zn0, Zn5, Zn10, and Zn Tab) consumption, breastfeeding, consumption of local foods, and number of LNS servings per day Caregiver: Type of food offered	Full day	Event– interval based	Quantitative
Sarma [57]	Bangladesh	6–59	Child: MNP ^3^ consumption	Full day	Event based	Qualitative
Langlois [52]	Burkina Faso	6–23	Meal: Preparation of the supplement, hygiene during preparation, and consumption of supplement leftovers Child: CSB+ w/oil ^4^, CSWB w/oil ^5^, SC+ ^6^, RUSF ^7^ consumption patterns	Full day	Event– interval based	Quantitative
Flax [55]	Malawi	6–17	Meal: Who fed the child and utensils used to feed Child: How many bites were offered and consumed of complementary foods and an FS ^8^	Full day	Event based	Quantitative
Flax [50]	Malawi	6–14	Meal: Supplement sharing behaviors, supplement leftover amount (CSB ^9^, LNS ^10^), location fed, person who fed child Child: Main position, number of bites offered, number of bites accepted, type of food consumed Caregiver: Hygiene behaviors before feeding, person responsible for feeding, utensils used to feed	Full day	Event based	Quantitative
Islam [56]	Bangladesh	8–11	Child: Intake of porridges with different energy densities (0.5, 1.0, or 1.5 kcal/g test meals)	Full day	Interval based	Quantitative
Kimmons [51]	Bangladesh	6–12	Caregiver: Food offered Child: Feeding frequency, meal types consumed, food consumed, breastfeeding duration	Full day	Interval based	Quantitative
Kamau-Thuita [48]	Kenya	0–24	Caregiver: Food preparation, feeding behaviors, washing clothes, bathing child, playing with child, holding child, and feeding behaviors Child: Resting and playing alone	Full day	Event based	Quantitative
Sawadogo [59]	Burkina Faso	6–24	Child: Types of foods consumed, number of breastfeeding and complementary feeding episodes, and amount of food consumed (grams)	Full day	Interval based	Quantitative
Wahid [58]	Indonesia	0–23	Child: Food consumed	Full day	Field notes	Qualitative
Rahman [45]	Bangladesh	6–24	Child: Feeding behaviors Caregiver: Hygiene/handwashing, cooking schedule, child food preparation, food storage type, food storage location, reheating and re-consumption practices, feeding behaviors	Full day	Field notes	Qualitative
Monterrosa [44]	Mexico	6–18	Caregiver: Household chores, child feeding, and childcare Child: Consumption of morning meal	Full day	Field notes	Qualitative
Kodish [27]	Kiribati	6–23	Child: Feeding behaviors Caregiver: Food preparation, food use, household food allocation, child feeding	Full day	Event– interval based	Qualitative
Kodish [24]	Mozambique	6–23	Caregiver: Food preparation, feeding behavior, and household food allocation Child: Feeding behavior and intake of SQ-LNS ^3^	Full day	Event– interval based	Qualitative
Kodish [23]	Malawi	6–23	Child: Dietary behavior, SQ-LNS ^2^ consumption Caregiver: Food preparation and feeding practices	Full day	Event– interval based	Qualitative
Kodish [43]	Malawi and Mozambique	6–23	Child: consumption throughout the day	Full day	Event– interval based	Qualitative
Dhingra [46]	India	12–24	Child: Amount of a standardized test meal consumed (nestum mixed in lactogen, milk, puffed rice, banana, and bread) using visual estimation of portion size, type of food offered, food spillage, and actual intake for every feeding episode	Full day	Event	Quantitative
Bonvecchio [47]	Mexico	6–23	Child and Caregiver: Supplement (i.e., papilla ^11^) usage as part of a conditional transfer program to assess compliance of three key intervention messages	Full day (6 h)	Event based	Quantitative
Paul [61]	Zanzibar	10–15	Child: Acceptability of instant soy rice without and with milk powder and corresponding consumption of the supplement	Full day	Event based	Mixed methods
Thakwalakwa [54]	Malawi	15	Child: Estimated energy intake based on food weights	Full day	Interval based	Quantitative
Iuel-Brockdorf [49]	Burkina Faso	6–23	Child: Consumption of six different corn–soy blended flours and six different LNS ^10^ with different amounts of milk and soy Caregiver: Use of LNS ^10^ leftovers including sharing and storage	Full day	Event based	Quantitative
Blaney [60]	Gabon	6–59	Child: Food consumption Caregiver: Food preparation, active complementary feeding, breastfeeding, household hygiene, hygiene practices related to children < 5 years, and women’s workload	Full day	Event based	Quantitative

^1^ Target behaviors: The list of key target behaviors provided in this table is not exhaustive but contains key highlighted behaviors from the manuscript. ^2^ SQ-LNS: small quantity lipid nutrient supplements, and SQLNS with different amounts of Zinc: Zn0, 0 mg; Zn5, 5 mg; Zn10, 10 mg; and ZnTab, LNS with no Zinc and a 5 mg Zinc tablet. ^3^ MNP: multiple micronutrient powder, multiple micronutrient powders. ^4^ CSB+ w/oil: corn soy blend plus with oil. ^5^ CSWB w/oil: corn soy whey blend with oil. ^6^ SC+: super cereal plus. ^7^ RUSF: ready-to-use supplementary food. ^8^ FS: fortified spread. ^9^ CSB: corn soy blend. ^10^ LNS: lipid nutrient supplement. ^11^ Papilla: nutritional supplement, powdered milk, sugar, and maltodextrine, distributed in a packaged, powdered form through the Oportunidades Program in Mexico.

**Table 3:** Studies employing indirect observations to study infant and young child feeding behaviors in LMICs.

Primary Author	Country	Child Age	Key Target Behaviors ^1^	Behavioral Recording Approaches	Analytic Approaches
Isanaka [9]	Niger	6–59	Child: RUTF ^2^ consumption Other: supplement condition and storage practices	Event based	Quantitative
Abbeddou [53]	Burkina Faso	11–16	Child: daily SQ-LNS ^3^ disappearance rate, number of days SQ-LNS/zinc capsule ^3^ consumed	Event based	Quantitative
Kodish [43]	Mozambique	6–23	Child: SQ-LNS ^3^ consumption	Event based	Quantitative
Ip [62]	Bangladesh	6–24	Child: sprinkle ^4^ consumption	Event based	Quantitative
Jefferds [28]	Kenya	6–59	Child: sprinkle ^4^ consumption	Event based	Quantitative
Adu-Afarwuah [42]	Ghana	6–23	Child: LNS-20 gM ^5^ consumption	Event based	Quantitative

^1^ Target behaviors: The list of key target behaviors provided in this table is not exhaustive but contains key highlighted behaviors from the manuscript. ^2^ RUTF: ready to use therapeutic foods. ^3^ SQ-LNS: small quantity lipid nutrient supplements. ^4^ Sprinkles: micronutrient powder. ^5^ LNS: lipid nutrient supplements.

## Data Availability

Data are contained within the article.
